# Investigating the time to blood culture positivity: why does it take so long?

**DOI:** 10.1099/jmm.0.001942

**Published:** 2025-01-06

**Authors:** Kerry Falconer, Robert Hammond, Benjamin J. Parcell, Stephen H. Gillespie

**Affiliations:** 1 Division of Infection and Global Health, School of Medicine, University of St Andrews, St Andrews, UK; 2 Ninewells Hospital and Medical School, Dundee, UK

**Keywords:** blood culture, bloodstream infection, rapid diagnostics

## Abstract

**Introduction.** Bloodstream infections (BSIs) are one of the most serious infections investigated by microbiologists. However, the time to detect a BSI fails to meet the rapidity required to inform clinical decisions in real time.

**Gap Statement.** Blood culture (BC) is considered the gold standard for diagnosing bloodstream infections. However, the time to blood culture positivity can be lengthy. Underpinning this is the reliance on bacteria replicating to a high concentration, which is necessary for the detection using routine blood culture systems. To improve the diagnosis and management of patients with BSIs, more sensitive detection methods are required.

**Aim.** The study aimed to answer key questions addressing the delay in BSI detection and whether the time to BSI detection could be expedited using a Scattered Light Integrated Collection (SLIC) device.

**Methodology.** A proof-of-concept study was conducted to compare the time to positivity (TTP) of Gram-negative BCs flagging positive on BacT/ALERT with an SLIC device. An SLIC device was utilized to compare the TTP of the most prevalent BSI pathogens derived from nutrient broth and BC, the influence of bacterial load on TTP and the TTP directly from whole blood. Additionally, the overall turnaround time (TAT) of SLIC was compared with that of a standard hospital workflow.

**Results.** Most pathogens tested took significantly longer to replicate when derived from BC than from nutrient medium. The median TTP of Gram-negative BC on BacT/ALERT was 13.56 h with a median bacterial load of 6.4×10^9^ c.f.u. ml^−1^. All pathogens (7/7) derived from BC at a concentration of 10^5^ c.f.u. ml^−1^ were detectable in under 70 min on SLIC. Decreasing *Escherichia coli* BC concentration from 10^5^ to 10^2^ c.f.u. ml^−1^ increased the TTP of SLIC from 15 to 85 min. Direct BSI detection from whole blood on SLIC demonstrated a 76% reduction in TAT when compared with the standard hospital workflow.

**Conclusion.** An SLIC device significantly reduced the TTP of common BSI pathogens. The application of this technology could have a major impact on the detection and management of BSI.

## Introduction

Bloodstream infections are life-threatening and time-sensitive [1,2]. However, the time to detect positive blood culture (BC) is lengthy and presents a major challenge in managing patients effectively [3,5]. The diagnosis of bloodstream infection (BSI) requires confirmation of viable bacteria in the bloodstream [6]. As the circulating levels of bacteria in patients with BSI are typically very low (1–100 c.f.u. ml^−1^), the confirmation of infection is reliant on bacterial replication *in vitro* [7,8]. A blood volume of 20–30 ml is recommended to accurately detect BSI with two positive BCs from two different body sites recommended for a diagnosis [6]. Currently, clinical laboratories employ automated blood culturing systems, such as BACTEC, BacT/ALERT and VersaTrek to detect BC positivity. Automated blood culturing systems are normally dependent on the active metabolism of bacteria and detect the presence of bacteria either by detecting increasing levels of CO_2_ by changes in pH and utilization of carbohydrate substrates by fluorescence intensity or by measuring pressure changes via O_2_ consumption and production of other gases (CO_2_, N_2_ and H_2_) [9,11]. The time to positivity (TTP) using these platforms ranges from 10 to 19 h and requires up to 5 days to confirm BC negativity [5,12,15].

The typical presentation and symptoms of BSI such as fever, chills, low blood pressure, increased heart rate and fatigue overlap with a range of other illnesses and make confirmation by culture essential [16]. Prompt, optimal antimicrobial therapy is of critical importance in BSI to resolve the infection early and prevent the onset of systemic inflammatory syndrome and sepsis [17]. The need for prompt optimal antimicrobial therapy in BSI is, however, complicated by the time taken to reach the diagnosis and the ongoing global rise in antimicrobial-resistant infections [18,20]. Long turnaround time (TAT) for BC positivity and subsequent antimicrobial susceptibilities increase the patient’s vulnerability to misdiagnosis and antimicrobial treatment failures. This can lead to increased recovery times, longer hospital stays, worse health outcomes and heightened risk of mortality [21,26].

Central to improving the BSI diagnostic pathway is the development and adoption of technologies that can quickly detect a BSI and inform treatment within a clinically useful timeframe. A Scattered Light Integrated Collection (SLIC) device was designed and developed to facilitate sensitive monitoring of bacterial growth and has been shown previously to detect clinically significant pathogens rapidly [27]. This patented technology is an innovative combination of laser light scattering, locked signal and an integrating detection space. This unique combination allows the detection of small changes in the bacterial population to be detected through the real-time monitoring of total light scatter. This paper reports on the use of SLIC to expedite BSI detection in comparison to an automated blood culturing platform and evaluates the ability of SLIC to perform direct detection of a BSI from whole blood.

## Methods

### Time to positivity of BC using BacT/ALERT

Samples submitted to the microbiology service of the Ninewells Hospital were collected prospectively between 17 January 2019 and 9 May 2019. BCs on the BacT/ALERT system that signalled positive and were confirmed to be Gram-negative bacteria by Gram stain were included in the study. Positive BCs were flagged on the BacT/ALERT system when an increase in CO_2_ production occurred, an indicator of microbial growth. The BacT/ALERT system was checked regularly between 0730 and 2100. BCs have a maximum incubation period of 5 days unless a special request is made. The TTP on the BacT/ALERT system was recorded for all BCs.

### Quantifying the bacterial load of flagged positive BCs on the BacT/ALERT system

Serial dilution plates were performed for all Gram-negative BCs. c.f.u. counts were undertaken immediately after an aliquot of BC was available and were performed using an adapted version of the Miles and Misra method [28] [28]. Briefly, 1 ml of BC was serially diluted between 10^−1^ and 10^−10^, and the last eight dilutions were plated in triplicate on brain heart infusion (BHI) agar and incubated overnight at 37 °C in aerobic conditions. c.f.u. counts were recorded the following morning.

### Preparation of surrogate BC

Surrogate blood cultures (SBCs) were made using 8 ml of tryptic soy broth (TSB), 1 ml of defibrinated horse blood (HB035, TCS Biosciences) and 1 ml of bacterial culture. BCs were spiked with either *Escherichia coli* ATCC 25922, *Staphylococcus aureus* ATCC 29213, *Klebsiella pneumoniae* ATCC 700603, *Pseudomonas aeruginosa* ATCC 27853, *Acinetobacter baumannii* ATCC 19606, *Streptococcus agalactiae* ATCC 12386 or *Streptococcus pneumoniae* ATCC 49619. A 1:10 blood–broth ratio was used to limit the antibacterial effects of blood in line with the UK Standards for Microbiology Investigations guidelines [29]. A final bacterial concentration of 10^5^ c.f.u. ml^−1^ was used to spike all BCs. The bacterial concentration was confirmed by optical density (OD_600_) and c.f.u. plate counts.

### The principle of SLIC

The SLIC method (v7) has been developed to capture total light scatter in real-time enabling high-resolution monitoring of bacterial populations. Cells undergoing replication, entering the stationary phase or cell death are recorded through changes in light scatter. The change in light scatter is detected by photodetectors within the integrated space and expressed in millivolts (mV). In the 6-well prototype used in this study, the unique scattering patterns were analysed across six wells simultaneously for each experiment. A detailed technical description of the SLIC device is provided in the following patents: GB201502194D0, GB201619509D0 and EP3759464A1. The patents are freely accessible online.

### Measuring bacterial generation time and time to detection of BSI pathogens derived from BC and nutrient culture using SLIC

To understand how the time to BC positivity could be improved, SBCs spiked with different BSI pathogens were prepared as described above and were analysed on SLIC. The growth behaviours of these bacteria were studied in both the presence and absence of blood. The bacteria were recovered from BC using a low-speed serum separation tube (LS-SST) method as previously described [30]. The recovered bacteria were diluted 1:10 into fresh pre-warmed TSB to achieve a final bacterial concentration of 10^5^ c.f.u. ml^−1^. Bacterial cultures were performed in triplicate on SLIC and analysed for 120 min. Bacterial growth was measured by total light scatter (mV) every second at 37 °C. Cultures of BSI pathogens grown in BHI media were also monitored on SLIC to determine the impact of blood on the bacterial generation time. Overnight culture was grown to a bacterial concentration of 10^8^ c.f.u. ml^−1^, confirmed by an OD reading of 1.00 and serially diluted to achieve a final bacterial concentration of 10^5^ c.f.u. ml^−1^ in pre-warmed BHI media on SLIC. Each SLIC run was performed for 60 min and was run in triplicate. Each run included three negative controls containing media only and three seeded cultures at a bacterial concentration of 10^5^ c.f.u. ml^−1^.

### The impact of bacterial concentration on the time to detection using SLIC

To measure the impact of bacterial load on the time to BC positivity, SBCs were spiked with different concentrations of *E. coli* and analysed directly on SLIC. *E. coli* ATCC 25922 was used as a model organism and was grown overnight from glycerol stock in 10 ml TSB and incubated at 37 °C. OD readings confirmed a bacterial concentration of 10^8^ c.f.u. ml^−1^ (OD 1.00). The bacterial culture was serially diluted to achieve a final concentration range of between 10^2^ and 10^5^ c.f.u. ml^−1^ in BC. The bacterial concentration was confirmed by c.f.u. using BHI plate counts. Bacteria were recovered from BC using the LS-SST method as previously described [30]. The recovered bacteria were diluted 1:10 into pre-warmed TSB media, and bacterial growth was monitored on SLIC for 120 min. Three biological repeats were performed for each BC concentration. Each run included three negative controls containing media only and three seeded cultures at one of the following bacterial concentrations: 10^2^, 10^3^, 10^4^ or 10^5^ c.f.u. ml^−1^.

### Direct detection of bacteria from whole blood using SLIC

To determine whether the detection of bacteria directly from a patient blood sample was achievable using SLIC, whole blood donated by healthy volunteers was spiked with very low concentrations of *E. coli*, one of the most prevalent BSI pathogens. Blood was donated by healthy volunteers within the School of Medicine at the University of St Andrews. A total of 40 ml of blood was taken at each blood donation by trained personnel. Blood was collected in a 10-ml ED vacutainer EDTA (K2) tube using an ED Vacutainer Safety-Lok blood collection set (Medisave, UK). Blood was stored at 4–6 °C until needed and was stored for a maximum of 6 days.

*E. coli* ATTC 29522 was grown overnight from glycerol stock in 10 ml TSB and incubated at 37 °C. OD readings were used to confirm a bacterial concentration of 10^8^ c.f.u. ml^−1^ (OD 1.00). The bacterial culture was then diluted accordingly to achieve a final concentration range of between 1 and 1000 c.f.u. ml^−1^ in whole blood. The final spiked bacterial concentration was confirmed by c.f.u. using BHI plate counts as previously described [28]. Three different blood volumes were tested: 2 ml, 4 ml and 8 ml to mimic blood volumes used in automated blood culturing platforms.

The spiked blood was transferred to an 8.5-ml BD Vacutainer SST II Advance tube (Fisher Scientific, UK) containing 1 mg l^−1^ of polyantholesulfonic acid sodium salt (Sigma-Aldrich, Cat No: 444464, UK). The red blood cells were removed from the sample by LS-SST centrifugation as previously described [30]. The supernatant of the sample (serum) was transferred and diluted 1:50 using pre-heated TSB media and placed directly into SLIC. Each spiked sample was run in triplicate, and each run consisted of three negative controls (unseeded serum).

### Comparison of standard of care and SLIC workflow

The time elapsed between sample collection and loading on the BacT/ALERT (transfer time) and the TTP of the BacT/ALERT system was measured for 79 *E. coli* BCs to calculate the mean TAT for the standard workflow. The TAT for SLIC was calculated using the maximum TTP measured in the study along with the processing times using the LS-SST recovery method.

### Data analysis

The TTP of the BacT/ALERT system and the bacterial concentration in the flagged positive BC bottle are expressed as median with interquartile range (IQR). Data grouped by bacterial species is expressed as mean±sem. Data generated by SLIC are expressed as mean±sem. The TTP of SLIC was defined as the time taken to achieve a clear differentiation between the positive and negative control on SLIC (when two consecutive timepoints were above the baseline of the negative control). The TTP of SLIC is expressed as mean±sem.

All data analysis was performed using GraphPad Prism v9.3.0.

### Data availability

Raw data were generated at the Ninewells Hospital, Dundee, and the University of St Andrews. Derived data supporting the findings of this study are available from the corresponding author (KF) on request.

## Results

### Time to positivity and bacterial load of Gram-negative BCs on the BacT/ALERT system

**Fig. 1.**
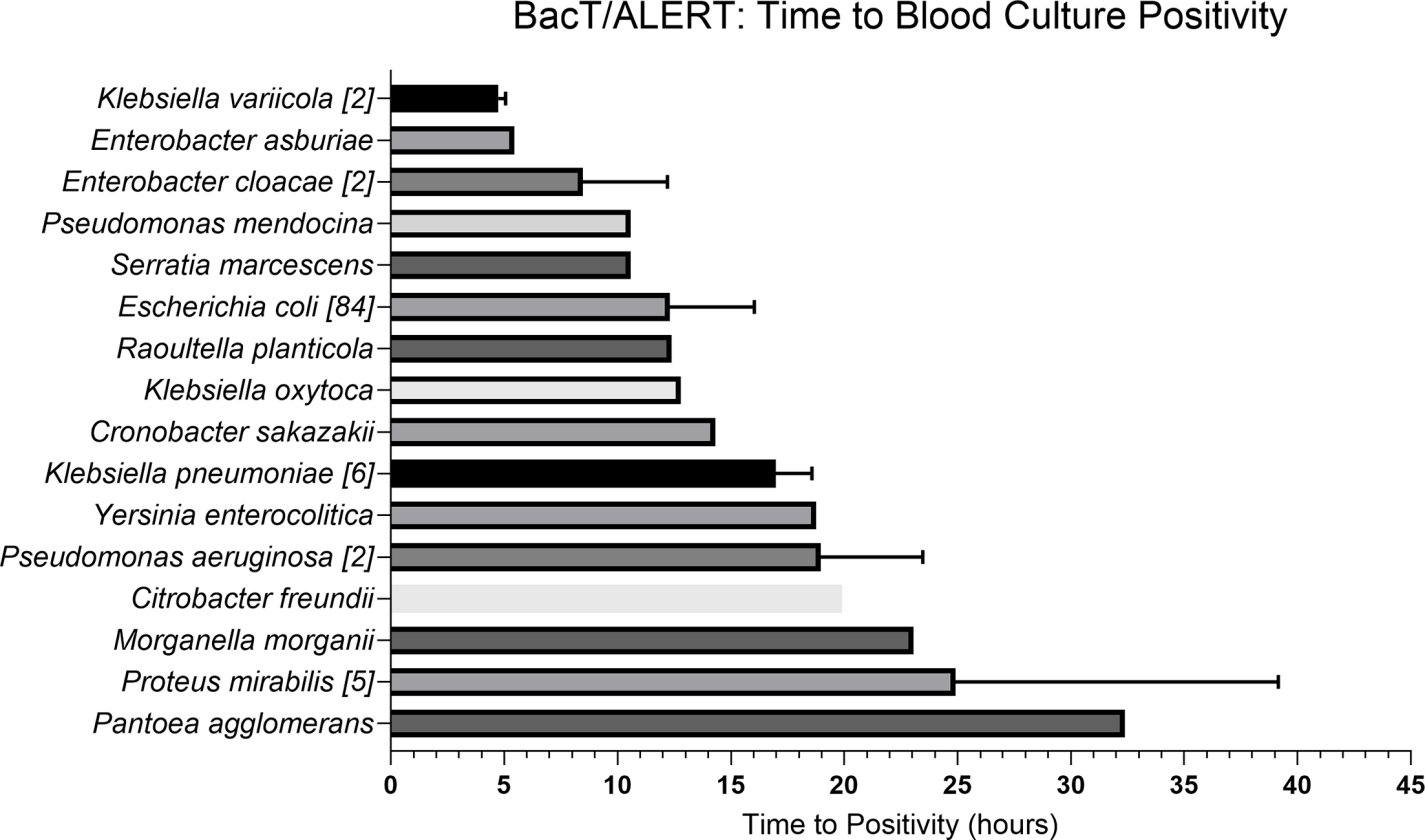
The detection time (hours) of Gram-negative BSI using BacT/ALERT 3D system. A total of 111 Gram-negative BCs flagged positive on the BacT/ALERT system over the 5-month study with a median TTP of 13.56 h (IQR, 10.57–19.66). The TTP varied with bacterial spp. For bacteria spp. detected more than once, the number of cases is given [n] with the mean±sem.

**Fig. 2.**
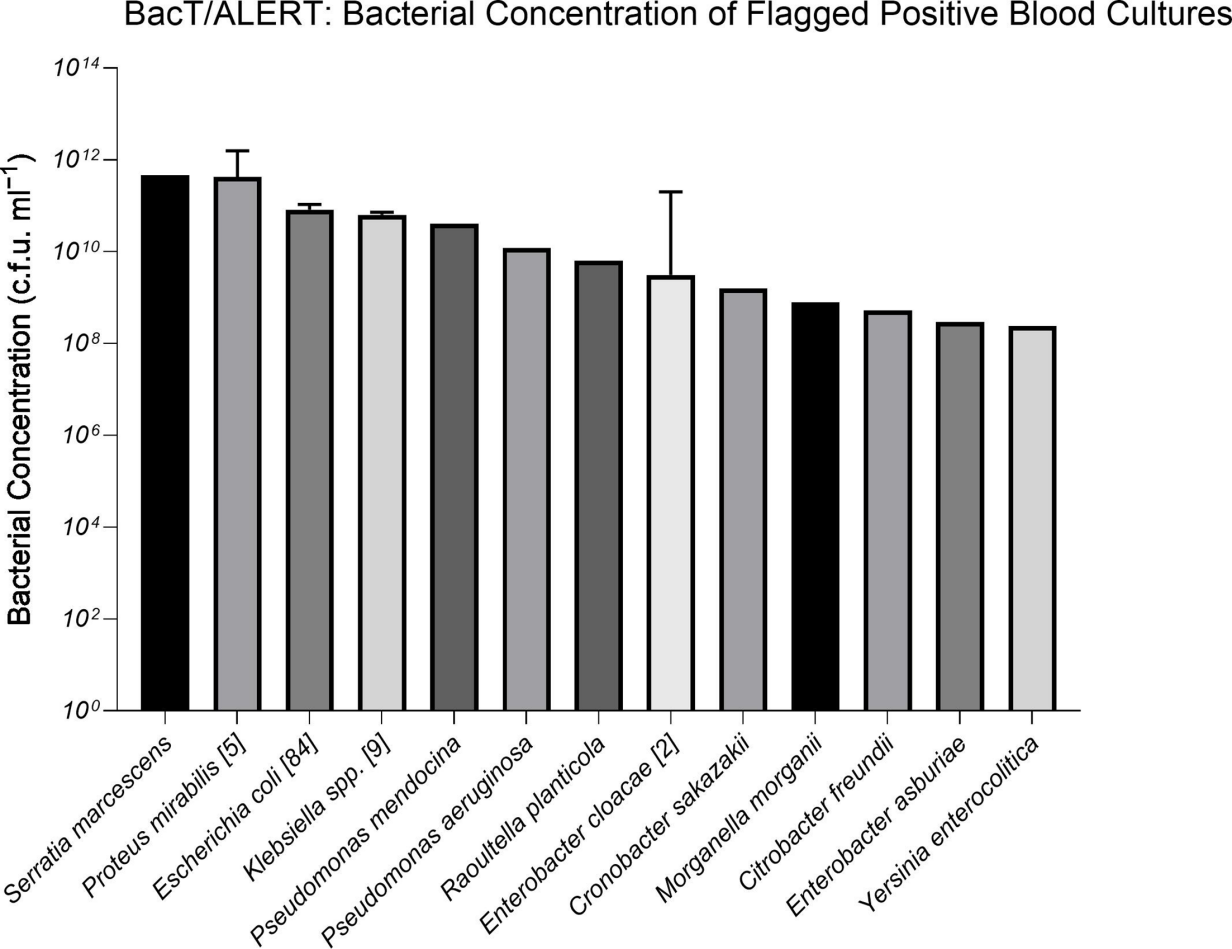
Quantifying the bacterial concentration of BCs that had flagged positive with Gram-negative bacteria. The median bacterial concentration for 109 Gram-negative BCs was 6.4×10^9^ c.f.u. ml^−1^ (IQR, 6.6×10^8^–7.26×10^10^). Two BCs, one positive for *P. aeruginosa* and another for *Pantoea agglomerans*, could not be quantified due to insufficient growth. For bacteria spp. detected more than once, the number of cases is given [n] with the mean±sem.

A total of 111 Gram-negative BCs flagged positive on the BacT/ALERT system in a median time of 13.56 h (IQR, 10.57–19.66) and varied with Gram-negative bacteria spp. (Fig. 1). The median Gram-negative bacterial concentration of flagged positive BCs was 6.4×10^9^ c.f.u. ml^−1^ (IQR, 6.6×10^8^–7.26×10^10^) (Fig. 2).

### The impact of blood on bacterial generation time

**Fig. 3.**
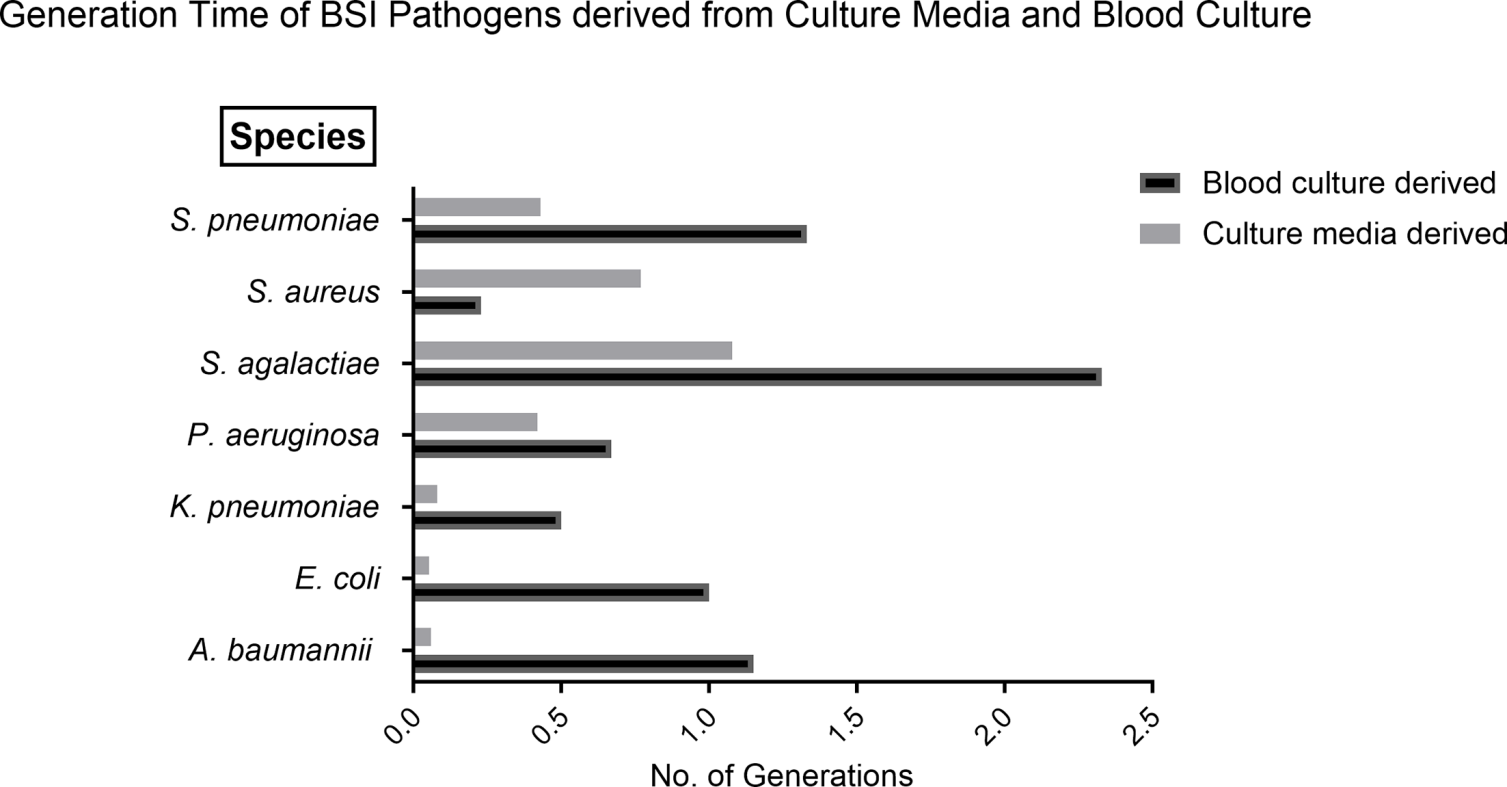
Comparison of TTP of different bacterial species grown in BC compared with BHI medium. TTP was defined as the time taken to achieve a clear differentiation between the positive and negative control on SLIC. The time to detection on SLIC was normalized by previously published generation times (Table S1, available in the online Supplementary Material). The TTP on SLIC was noticeably longer when bacteria were grown in BC compared with media except for *S. aureus*. All BSI pathogens were detectable on SLIC in under 60 min apart from *S. agalactiae* (70 min). The starting bacterial concentration was 10^5^ c.f.u. ml^−1^.

After studying the TTP and bacterial load of positive BCs on the BacT/ALERT system, the next series of experiments looked more closely into the processes that underpin BC examination (Fig. 3). The increased sensitivity of SLIC provided an insight into bacterial growth rate when inoculated in culture media compared with BC. The TTP on SLIC was significantly longer for bacteria sub-cultured from BC compared with culture media except for *S. aureus*. SLIC enabled most bacteria (6/7) to be detectable in less than 60 min and within two bacterial replication cycles. The exception to this was *S. agalactiae,* which required greater than two generations and was detectable within 70 min.

### The impact of bacterial load on time to BC positivity on SLIC

**Fig. 4.**
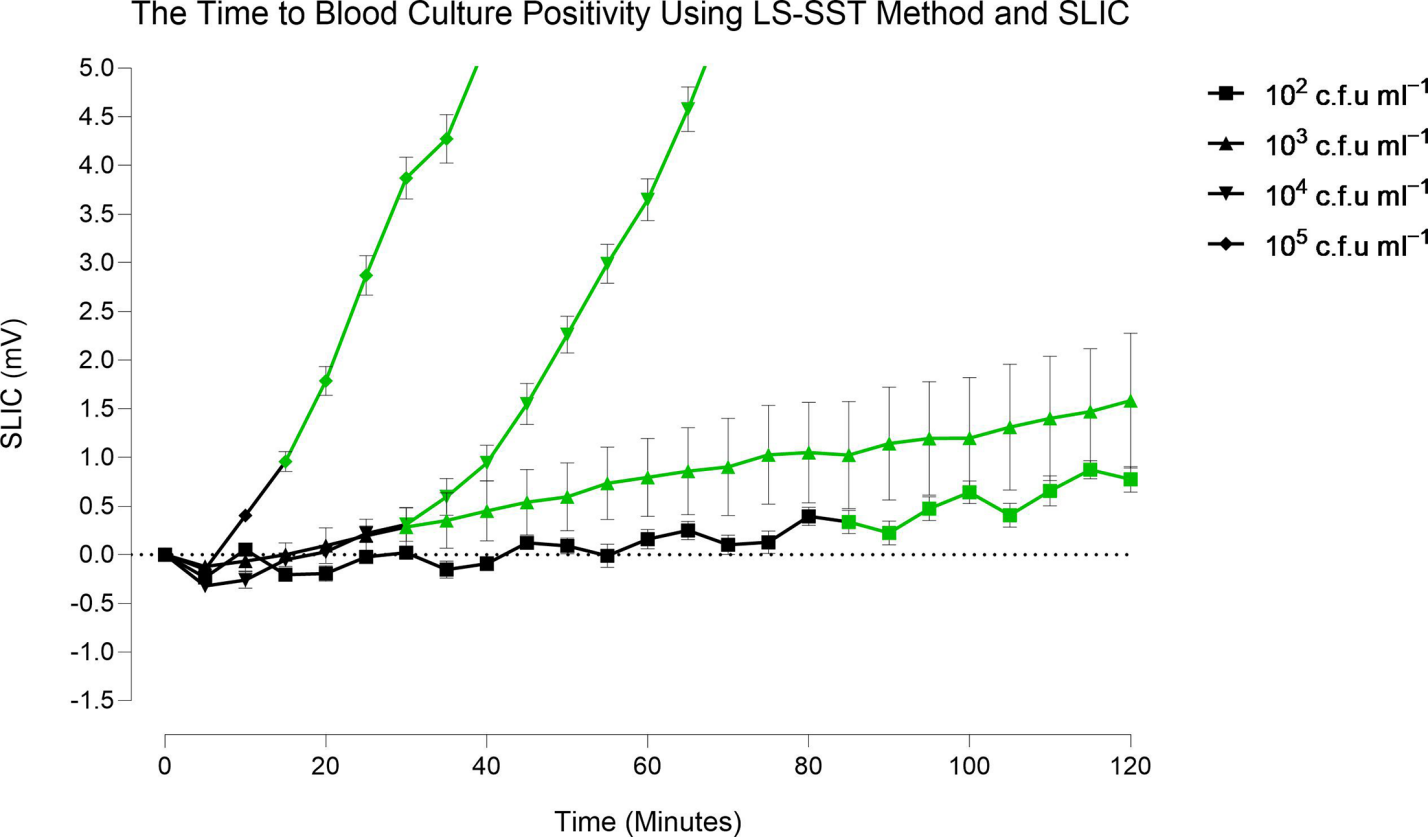
*E. coli* BCs spiked with four different concentrations were examined on SLIC over 120 min. The LS-SST method was used to recover bacteria from BC, and the bacteria were grown in pre-warmed TSB media for SLIC analysis. The TTP was determined when the mV output deviated from the baseline consistently for two timepoints (indicated in green). The baseline was defined by the negative control mV output (not shown on graph). A total of three biological repeats were performed at each BC concentration, the mean±sem is plotted. A concentration time-dependent relationship was established for all BCs. BCs spiked with *E. coli* at 10^5^, 10^4^ and 10^3^ c.f.u. ml^−1^ were detectable within 15 to 30 min, respectively. In comparison, BC spiked with *E. coli* at 10^2^ c.f.u. ml^−1^ was detectable within 85 min.

As one of the most prevalent BSI pathogens (20–30 % of cases worldwide), *E. coli* was used as a model pathogen to explore the impact of starting bacterial load on the time to BC positivity on SLIC. The time to BC positivity was measured for four different concentrations of *E. coli* (10^2^–10^5^ c.f.u. ml^−1^) over 120 min (Fig. 4). The most concentrated BC separated from the baseline almost immediately and reached exponential growth within 15 min. The lower concentrated BC demonstrated a steady increase in growth with small increases in mV readings on SLIC. At the lowest bacterial concentration of 10^2^ c.f.u. ml^−1^, separation from the baseline occurred within 85 min.

### Direct detection of bacteria from spiked whole blood using SLIC

**Fig. 5.**
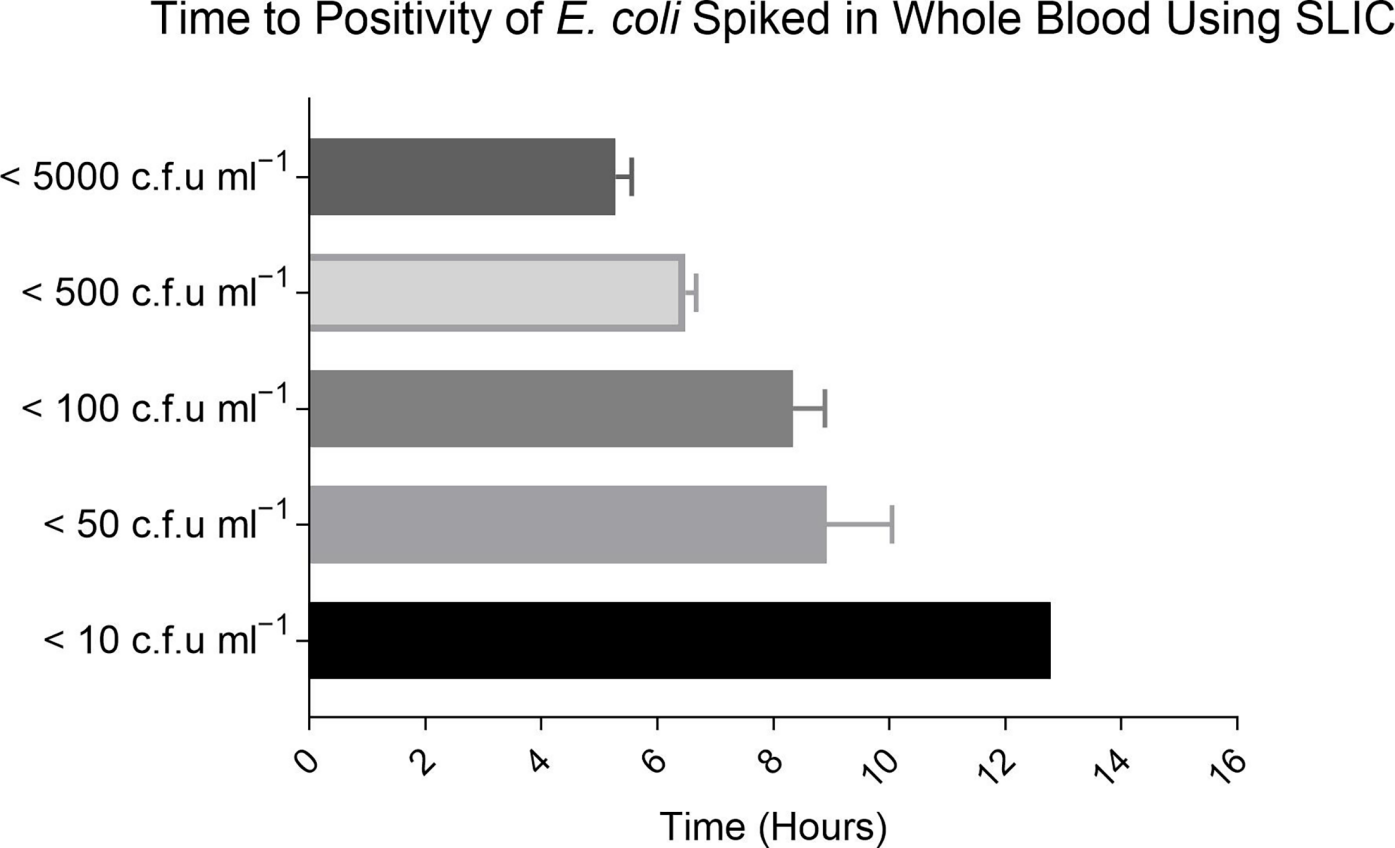
The TTP of *E. coli* on SLIC directly from whole blood. The mean TTP for a bacterial concentration range of 10–10^3^ was 8.54±0.99 h, 6.48±0.81 h and 6.48±0.67 h for the blood volumes of 2 ml, 4 ml, and 8 ml respectively. The overall mean TTP across all bacterial concentrations and blood volumes was 7.16±0.82 h.

Having established the TTP from BC on SLIC, the study progressed to explore the direct detection of BSI from whole blood. *E. coli* was used as a model organism for these series of experiments. The LS-SST recovery method supported the direct detection of *E. coli* from whole blood. A concentration–time-dependent relationship was established with the highest bacteria load of 10^3^ c.f.u. ml^−1^ detectable within 5 h and associated with the shortest TTP on SLIC (Fig. 5). The TTP did not significantly vary with blood volume when the bacterial concentration was above 10 c.f.u. ml^−1^. However, a bacterial load of <10 c.f.u. ml^−1^ was only detectable on SLIC when using the lowest blood volume of 2 ml with a TTP of 12.77 h.

### Time to detection of an *E. coli* BSI using the SLIC method compared with the BacT/ALERT workflow

**Fig. 6.**
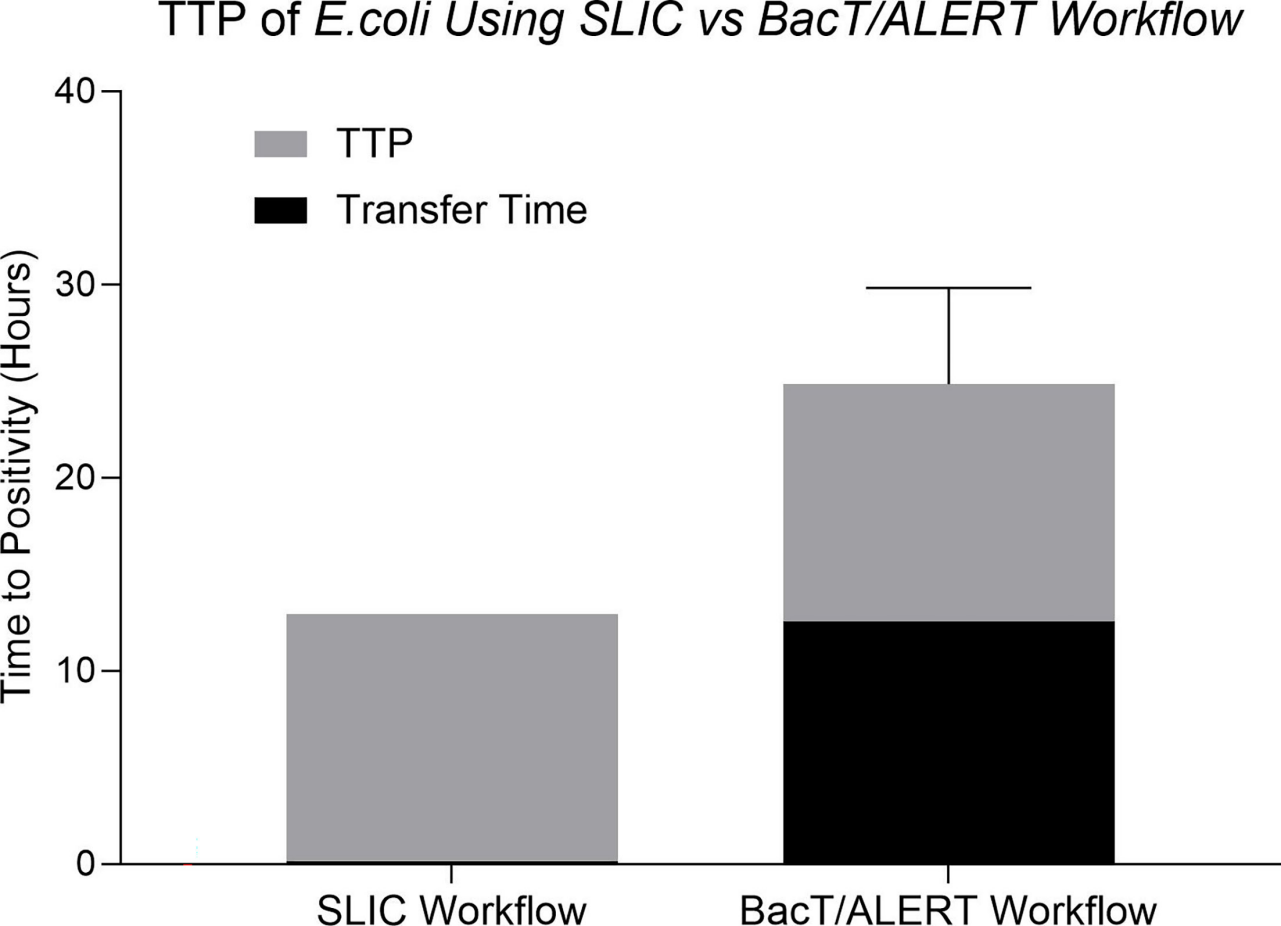
Time saving of the SLIC workflow against the BacT/ALERT workflow. The SLIC workflow involves the direct processing of whole blood with a 10-min serum separation step before the sample is ready for use in SLIC. The analysis of TTP on SLIC directly from whole blood was comparable to BacT/ALERT for the detection of *E. coli*. However, by directly sampling from whole blood (patient sample), the mean transfer time of 12.54±1.28 h was nullified and could potentially enable BC positivity to be confirmed within the same day as sampling. The slowest meantime for detection on SLIC (*E. coli* at <10 c.f.u. ml^−1^) was taken as an illustrative example. Depending on blood volume and starting bacterial concentration, the SLIC workflow may be quicker than indicated.

To determine whether the direct processing of blood samples on SLIC would accelerate the time to the detection of an *E. coli* BSI, the SLIC TAT was directly compared with the standard workflow in a clinical setting. The longest TTP on SLIC of 12.77 h was associated with the smallest blood volume of 2 ml and the lowest bacterial concentration of <10 c.f.u. ml^−1^. For comparison, the mean TTP of *E. coli* BC on the BacT/ALERT system was 12.30±3.7 h. The TAT to confirm an *E. coli* BSI was 24.84±4.98 h using the standard BacT/ALERT workflow in comparison to the SLIC workflow with a maximum TAT of 12.94 h (Fig. 6).

## Discussion

The detection of BSI is one of the most important clinical investigations conducted by a clinical microbiology laboratory [10]. Cultivation of pathogens from the bloodstream remains the gold standard for the diagnosis of BSI [6]. Conventionally, the time taken to culture bacteria from the bloodstream is far greater than the time required for bacteria to replicate. A method, with a limit of detection close to that of the circulating levels found in a BSI patient (1–100 c.f.u. ml^−1^), is required [7,8].

In this study, we compared a commonly used automated blood culturing system with an innovative light scattering technology for the detection of Gram-negative BSI. We demonstrated that TTP was possible in a median time of 13.56 h (IQR, 10.57–19.66) when measured for over 100 Gram-negative BCs on the BacT/ALERT platform. The long time to positivity is in keeping with other studies which report BC positivity within 10–19 h using various automated blood culturing platforms [5,12,15, 31].

The current drawback of BSI diagnostics is the large bacterial concentration required before automated systems signal positive. Quantifying the concentration of flagged positive BCs on BacT/ALERT demonstrated a limit of detection of 10^8^ c.f.u. ml^−1^. This agrees with other blood culturing systems such as the BACTEC FX which achieved similar detection sensitivities [32]. The limit of detection of these platforms is far greater than the bacterial concentration of 10^5^ c.f.u. ml^−1^ required for bacterial identification and antibiotic susceptibility testing [33]. Evidently, the generation time of bacteria and the associated production rate of growth metabolites, which many automated blood culturing systems rely on, do not allow detectable bacterial growth in a rapid timeframe. The high bacterial concentration required for BC positivity negatively impacts the time to detection and the timeliness of downstream analysis. A limitation that can be overcome by developing and employing more sensitive methods for monitoring bacterial growth, such as light scatter.

Quantifying the effect of blood on bacterial generation time is challenging. However, the sensitivity of SLIC technology allowed this to be possible, thereby bridging a current gap in our knowledge. For most BSI pathogens (6/7), the generation time was prolonged in the presence of blood. This observed suppression of bacterial growth may be a combination of many mechanisms and may be non-specific or specific to each bacterial species [34,38]. The findings confirm the widespread assumption of the antibacterial effects of blood on bacterial growth [39].

The most frequent BSI pathogens isolated in clinical samples are *E. coli* and *S. aureus* [6]. The generation time of *E. coli* was constant at 20 min, whereas *S. aureus* replicated up to three times faster in BC than in nutrient-rich media. This observation is supported by a study conducted by Lee and colleagues [40] who documented a noticeable growth enhancement in *S. aureus* in blood compared with blood-free media [40]. This may be explained by the ability of *S. aureus* to utilize haemoglobin as an iron source through an internal iron-regulated surface determinant system, which has been reported as an essential requirement for *S. aureus* pathogenesis in BSI [37,41]. Additionally, this may offer an explanation as to why the generation time of *S. aureus* was enhanced in the presence of blood over other BSI pathogens where the utilization of haemoglobin is not uniform across bacterial pathogens [35,36].

Among the Gram-negative bacteria, *E. coli* is a predominant BSI pathogen and is implicated in 20–30 % of BSI cases worldwide [6,42, 43]. As such, this pathogen was selected to study the TTP on SLIC directly from BC and in a bid to facilitate point-of-care testing, whole blood. A variety of bacterial concentrations and blood volumes were tested on SLIC to reflect the low bacterial loads reported in BSI and to mimic blood volumes used in automated BC systems. For both sample types, the TTP was dependent on the starting bacterial concentration. From BC, SLIC offered rapid detection at all bacterial concentrations tested and TTP ranged from 15 to 85 min. The direct detection of bacteria from whole blood demonstrated a lag in bacterial growth with positivity determined between 6 and 9 h for a concentration range of 10–100 c.f.u. ml^−1^. In comparison, *E. coli* BC positivity was detectable in 12.3±3.7 h on the automated BacT/ALERT System. This demonstrates a promising timesaving could be made from BC and directly from whole blood using SLIC.

Facilitating testing directly from the patient sample can have a significant impact on the time to diagnosis and treatment. In this study, the pre-laboratory phase was associated with a meantime of 12.54±1.28 h and significantly prolonged the time to BC positivity and downstream analysis. The direct detection of *E. coli* from whole blood using SLIC demonstrated a potential timesaving of 76% compared with the standard workflow. This comparison was based on the most implicated BSI pathogen, *E. coli*, and at a bacterial concentration commonly isolated from BSI patients [6,8,42, 43]. The significance of reducing time to BSI diagnosis has been strongly correlated with enhanced patient care, reduced mortality and morbidity and has motivated the need for faster BSI diagnostics [20,44].

The TTP reported from BC and whole blood using SLIC was representative of *E. coli* only. It is appreciated that in other patient populations and clinical settings, different pathogens will be prevalent. Therefore, to support the use of SLIC as a rapid diagnostic tool in BSI, a larger multi-centre study incorporating a wide range of BSI pathogens and clinical patient samples is needed. Polymicrobial samples represent a small percentage of positive BCs, and the TTP reported are representative of monomicrobial growth only. Further work is needed to assess the impact of multiple pathogens in blood on the TTP using SLIC. The current prototype of SLIC has the capacity to screen six blood samples at one time, and this currently limits the way SLIC testing could be conducted in a clinical setting. Many BCs are requested, but few are positive with a low BC positivity rate of between 7.5 and 14.1% [45,46]. Thereby, to achieve a sufficient number of positive samples, it is likely that a larger cohort study from whole blood would need to be performed retrospectively.

## Conclusion

In BSIs, rapid confirmation of bacterial growth will be valuable in the clinical decision-making of patient diagnosis and treatment. This is especially important for patients presenting with non-specific symptoms, a common occurrence in septic and BSI patients. The high sensitivity and the low limit of detection offered by SLIC supported a marked reduction in time to BC positivity over existing methods and made the direct detection of bacteria from BC and whole blood possible. The reduced number of steps to BSI detection and the advantages of phenotypic detection using SLIC including affordability, simplicity and rapidity presents a promising alternative BSI detection strategy supporting earlier diagnosis and better prognosis in BSI.

## supplementary material

10.1099/jmm.0.001942 Uncited Table S1.

## Abbreviations

BC: blood culture
BHI: brain heart infusion
BSI: bloodstream infection
IQR: interquartile range
LS-SST: low-speed serum separation tube
mV: millivolts
SBCs: surrogate blood cultures
SLIC: Scattered Light Integrated Collection
TAT: turnaround time
TSB: tryptic soy broth
TTP: time to positivity
