# Supplementary material for: Investigating the time to blood culture positivity: why does it take so long?
Source: J Med Microbiol. 2025 Jan 6;74(1):001942. doi: 10.1099/jmm.0.001942 (PMC11701752; doi:10.1099/jmm.0.001942)
Supplement: Uncited Table S1. [file jmm-74-01942-s001.pdf]

Supplementary Material

Table S1. List of bacterial spp. and published generation times under optimal conditions.

Organism	Estimated Generation Time (minutes)	Reference
S. aureus	20	Missiakas, D.M. and Schneewind, O. 2013. Growth and Laboratory Maintenance of *Staphylococcus aureus*. *Current Protocols in Microbiology*. 28: 9C.1.1-9C.1.9.
K. pneumoniae	40	Regué, M., Hita, B., and Piqué, N. 2004. A gene, uge, is essential for *Klebsiella pneumoniae* virulence. *Infection and Immunity*. 72(1):54-61
P. aeruginosa	45	Vallet-Gely, I and Boccard, F. 2013. Chromosomal organization and segregation in Pseudomonas aeruginosa. *PLoS Genetics*. 9(5):e1003492.
E. coli	20	Gibson, B., Wilson, D. J., Feil, E., et al. 2018. The distribution of bacterial doubling times in the wild. *Proceedings Biological sciences*. 285(1880):20180789.
A. baumannii	48	Moffatt, J.H., Harper, M., Harrison, P., et al. 2010. Colistin resistance in *Acinetobacter baumannii* is mediated by complete loss of lipopolysaccharide production. *Antimicrobial.*
S. agalactiae	30	Milligan, T.W., Doran, T.I., Straus, D.C., Mattingly, S.J. 1978. Growth and amino acid requirements of various strains of group B streptococci. *Journal of Clinical Microbiology*.
S. pneumoniae	30-35	Morlot, C., Zapun, A., Dideberg, O., et al. 2003. Growth and division of *Streptococcus pneumoniae*: localization of the high molecular weight penicillin-binding proteins during the cell cycle. *Molecular Microbiology*. 50: 845-855.
